# The role of neutrophils in osteosarcoma: insights from laboratory to clinic

**DOI:** 10.3389/fimmu.2024.1490712

**Published:** 2024-11-08

**Authors:** Ming Xia, Yu Han, Lihui Sun, Dongbo Li, Chunquan Zhu, Dongsong Li

**Affiliations:** Department of Orthopedics, The First Hospital of Jilin University, Changchun, Jilin, China

**Keywords:** osteosarcoma, neutrophils, tumor microenvironment, neutrophil extracelluar traps, TANs

## Abstract

Osteosarcoma, a highly aggressive malignant bone tumor, is significantly influenced by the intricate interactions within its tumor microenvironment (TME), particularly involving neutrophils. This review delineates the multifaceted roles of neutrophils, including tumor-associated neutrophils (TANs) and neutrophil extracellular traps (NETs), in osteosarcoma’s pathogenesis. TANs exhibit both pro- and anti-tumor phenotypes, modulating tumor growth and immune evasion, while NETs facilitate tumor cell adhesion, migration, and immunosuppression. Clinically, neutrophil-related markers such as the neutrophil-to-lymphocyte ratio (NLR) predict patient outcomes, highlighting the potential for neutrophil-targeted therapies. Unraveling these complex interactions is crucial for developing novel treatment strategies that harness the TME to improve osteosarcoma management.

## Introduction

Osteosarcoma is a primary malignant bone tumor characterized by the uncontrolled proliferation of osteoblastic cells, predominantly affecting children and adolescents (1). This aggressive cancer is associated with significant morbidity and mortality, necessitating a deeper understanding of its pathogenesis and progression (2).

The tumor microenvironment (TME) plays a pivotal role in the development and progression of cancer. TME encompassing a complex milieu of cellular and molecular components that interact with tumor cells and influence their behavior (3). In TME, various cell types, including immune cells, fibroblasts, endothelial cells, and extracellular matrix components, dynamically interact with tumor cells to create a supportive niche for tumor growth and dissemination (4). In osteosarcoma, the tumor microenvironment is characterized by an immunosuppressive milieu, driven by the secretion of cytokines, chemokines, growth factors, and extracellular matrix remodeling enzymes (5). These factors not only promote tumor cell proliferation, survival, and invasion but also modulate the immune response, angiogenesis, and metastatic potential of osteosarcoma (6).

Neutrophils are white blood cells that play a key role in the innate immune response to infection and inflammation (7). In recent years, evidence has highlighted the multifaceted roles of neutrophils in the TME of various cancers, including osteosarcoma (8). Neutrophils can be recruited to the TME in response to tumor-derived signals and inflammatory mediators, where they interact with tumor cells and other stromal components (9).

Neutrophils have long been recognized for their role in osteosarcoma. Initially, the prognostic significance of the neutrophil-to-lymphocyte ratio in osteosarcoma was identified (10). Subsequent research has further elucidated the involvement of neutrophils, particularly neutrophil extracellular traps (NETs) and tumor-associated neutrophils (TANs), in the immune microenvironment and progression of osteosarcoma (8).

While neutrophils can promote tumor progression in various cancers, the specific mechanisms and the extent of their influence may vary. In osteosarcoma, neutrophils may contribute more significantly to the immunosuppressive tumor microenvironment and the promotion of metastasis due to the unique interactions between neutrophils and the bone matrix, as well as the high propensity of osteosarcoma cells to metastasize to the lung and the roles of neutrophils in the body after surgery (5). Previous findings underscore the intricate involvement of neutrophils in the complex interplay within TME of osteosarcoma, highlighting their potential as key modulators and therapeutic targets for improving clinical outcomes in this aggressive bone cancer (11). In the context of osteosarcoma, neutrophils have been shown to interact uniquely with the tumor microenvironment (12). The osteosarcoma microenvironment is a complex ecosystem that includes not only the tumor cells but also a variety of immune cells, bone cells, extracellular matrix components, and signaling molecules (1). Neutrophils in this context can be affected by the tumor to promote tumor growth, survival, and metastasis (13).

**Figure 1:**
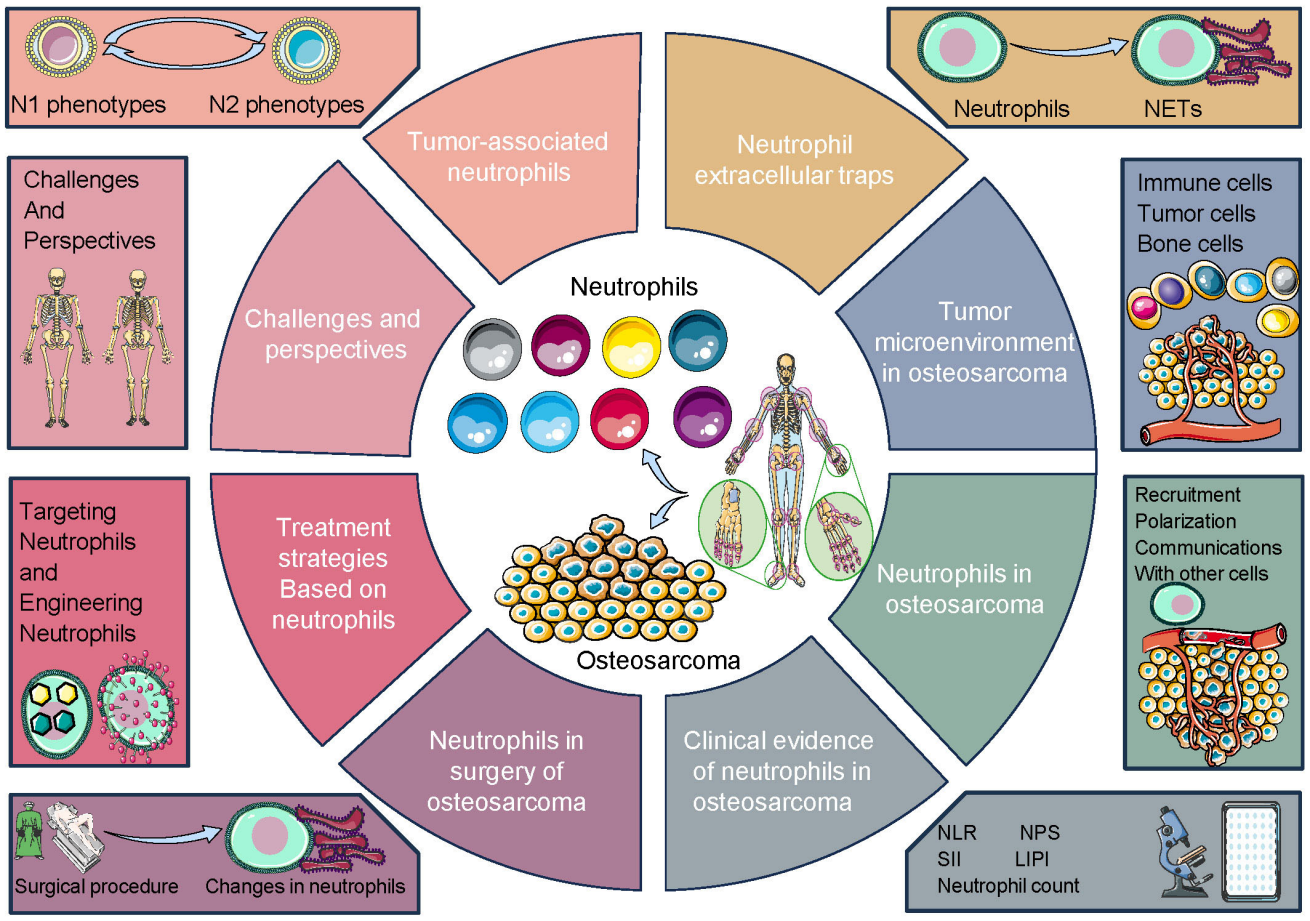
Overall design of our study. The nuanced interactions between neutrophils, specifically tumor-associated neutrophils (TANs) and neutrophil extracellular traps (NETs), with key osteosarcoma microenvironment (TME) constituents. It emphasizes the reciprocal modulation between TANs and TME cellular components, such as cancer-associated fibroblasts (CAFs) and regulatory T cells (Tregs), and molecular mediators including cytokines and chemokines, which orchestrate a pro-tumorigenic or tumor-suppressive milieu.

Understanding the interactions between TANs, NETs, and TME in osteosarcoma is essential for elucidating the mechanisms underlying tumor progression and identifying potential therapeutic targets. Targeting the immune cell components and inflammatory pathways within the tumor microenvironment may offer novel strategies for the treatment of osteosarcoma and improve patient outcomes. This review aims to provide insights into the multifaceted roles of neutrophils in osteosarcoma, spanning from fundamental laboratory research to potential clinical implications. By elucidating the intricate interactions between neutrophils and osteosarcoma, this review seeks to enhance our understanding of the complex tumor microenvironment and identify novel therapeutic strategies for the management of this aggressive bone cancer (**Figure 1**).

## TANs in the tumor

**Figure 2:**
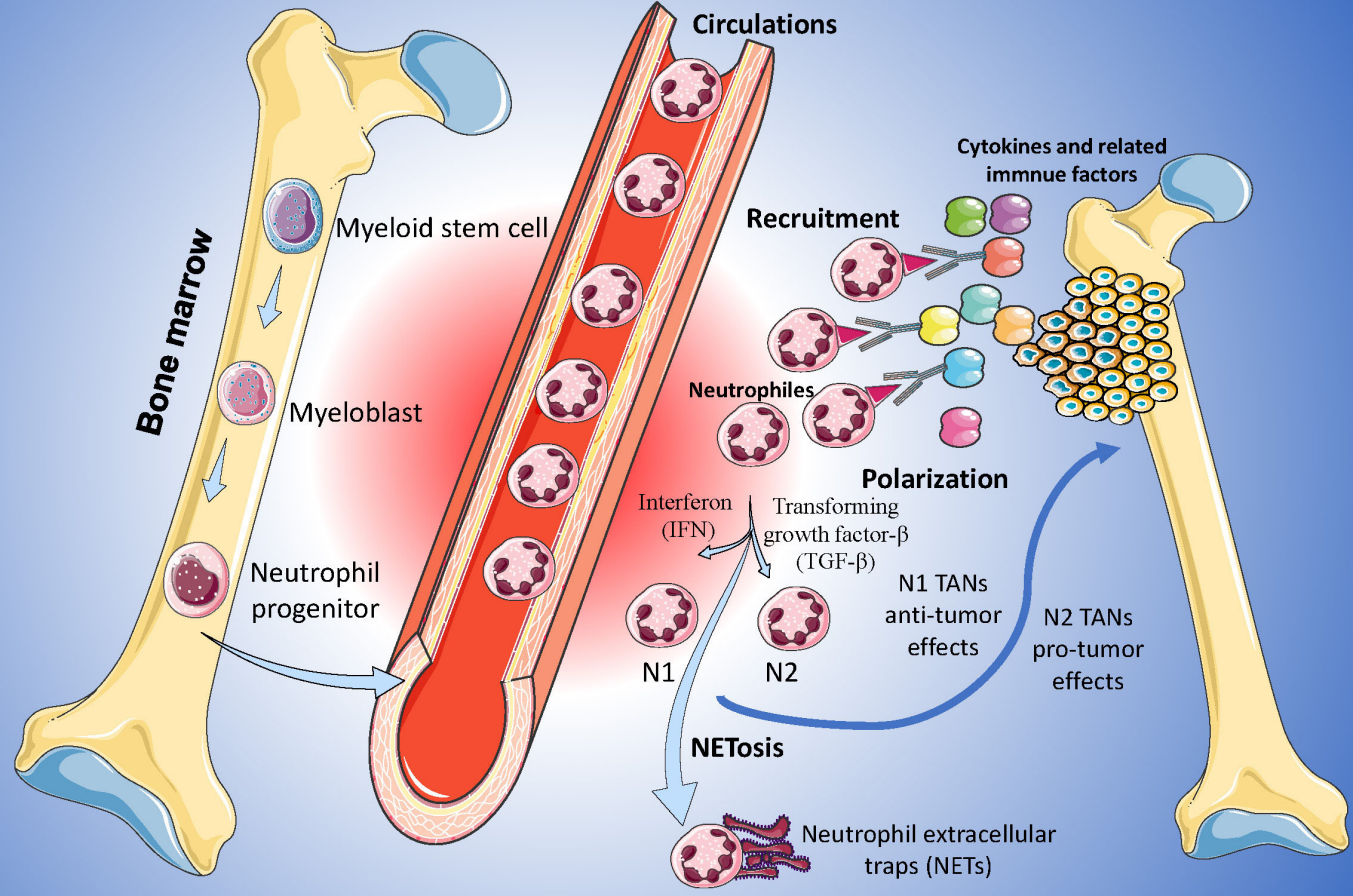
Function and roles of Tumor-Associated Neutrophils (TANs) in Osteosarcoma. N1 TANs, characterized by their anti-tumoral functions, are depicted engaging in ROS-mediated cytotoxicity and antigen presentation, while N2 TANs are portrayed as pro-tumorigenic, secreting factors such as MMP-9 and VEGF that facilitate angiogenesis and immunosuppression.

Neutrophils are the most abundant immune cells in the human body and constitute 50%-70% of all white blood cells (14). Due to the limited proliferation capacity and lifespan of neutrophils, the understanding of the roles of neutrophils in heterogeneous tumors has been lacking in recent decades (15). In recent years, owing to novel biotechnology, such as single-cell sequencing, there has been increasing attention on neutrophils in tumor-related research (16). Nowadays, the heterogeneity of TANs far exceeds the simple classification of several groups (17). According to their roles and functions in TME, TANs are classified into anti-tumor (N1) and pro-tumor (N2) phenotypes (**Figure 2**), and these two TANs phenotypes with opposing effects may regulate the initiation, proliferation, metastasis, and immune suppression (18).

Usually, neutrophils are continuously produced in the hematopoietic cords of the bone marrow and are regulated by transcription factors and proteins such as CCAAT-enhancer binding protein (CEBP/α) and colony-stimulating factors (19). In the tumor tissues, the function and polarization of neutrophils were mainly regulated by the factors in inflammatory TME. Different transcription factors and proteins may contribute to different neutrophil phenotypes (20). For example, interferon type 1 (IFN-1) may enhance the ability of adhesion and phagocytosis of neutrophils and force neutrophils to polarized towards N1 phenotype, and reversely, transforming growth factor-β (TGF-β) is a driver for N2 phenotype (21, 22). Furthermore, many other factors were also identified as the drivers of the polarization of neutrophils, including adenosine triphosphate, S100A9, adenosine, and so on (23, 24).

In recent years, some downstream pathways related to TANs were identified. N1 cell phenotypes usually perform their anti-tumor effects via reactive oxygen species (ROS) related pathways (25). ROS may up-regulate the superoxide-dependent Ca2+ channel of the tumor cells, contribute to the disorder of the Ca2+ levels, and lastly inhibit the development of the cancer (26). Reversely, tumor development may be upregulated by the factors produced by N2 phenotypes, including neutrophil elastase (NE) and matrix metalloproteinases (15).

In addition to immune regulations, TANs were also regulated by metabolic factors: the factors produced in the glucose metabolism, lipid metabolism, tricarboxylic acid cycle, and amino acid metabolism were able to reprogram the metabolism of TANs (7, 27). The oxygen deprivation TME may contribute to the glycolytic effects of TANs (28), and the factors produced in the glycolysis may inhibit the proliferation of movement of T cells and play their immunosuppressive functions (29). The TANs after metabolism reprogramming may contribute to a higher level of hypoxia-inducible factor 1 alpha (HIF-1α) expression (30). HIF-1α is a key transcription factor that plays a critical role in cellular responses to low oxygen levels, or hypoxia. In the context of tumors, HIF-1α is known to be a master regulator of the adaptive mechanisms that cancer cells employ to survive and proliferate under hypoxic conditions (31). Activation of HIF-1α in tumor cells leads to the upregulation of genes involved in angiogenesis, glycolysis, and cell survival, promoting tumor growth and metastasis (31, 32).

## NETs in tumor

In addition to neutrophils, neutrophil-related components have also attracted increasing attention. Neutrophil extracellular traps (NETs) are web-like structures composed of chromatin, histones, and antimicrobial proteins released by activated neutrophils in response to various stimuli, including infection, inflammation, and cancer (33). In the context of tumors, emerging evidence suggests that NETs play an important role in the tumor microenvironment (34). NETs have been implicated in promoting tumor progression by facilitating tumor cell adhesion, migration, and invasion, as well as inducing immunosuppression and angiogenesis (35).

The formation of NETs is not spontaneous but rather occurs abundantly in activated neutrophils (33). The formation of NETs is related to a distinct form of cell death, mediated by ROS and termed neutrophilic inflammatory cell death (NETosis), distinguishing it from traditional apoptosis and necrosis (36). Upon stimulation by extracellular physicochemical factors, the chromatin within the nucleus undergoes abnormal changes, and enzymes within cytoplasmic vesicles are activated, ultimately leading to the rupture of the cell membrane and release of the contents (37). In certain instances, neutrophils do not need to sacrifice themselves to release NETs, as non-lytic NETosis can occur during Staphylococcus aureus infection. In this scenario, neutrophils rapidly release chromatin extracellularly and undergo degranulation to release various enzymes, thereby forming extracellular NETs (33).

Initially, the roles of NETs in cancer metastasis, especially in the premetastatic niche, were recognized. NETs may facilitate tumor cell migration and invasion by releasing pro-inflammatory cytokines and chemokines that attract tumor cells to the site of NETs deposition, including IL-1, IL-6, IL-8, and so on, promoting their movement toward distant sites (38). Additionally, NETs induce an epithelial-mesenchymal transition (EMT) in cancer cells, leading to the acquisition of a mesenchymal phenotype that enhances their migratory and invasive abilities (39). Furthermore, NETs interact with endothelial cells, causing a loss of cell-to-cell junctions and altering the morphology of the endothelium, facilitating tumor cell intravasation and extravasation (40). By capturing circulating tumor cells and creating a permissive environment in pre-metastatic and metastatic niches, NETs promote the establishment and growth of metastatic lesions (41).

Nowadays, studies have proven that NETs also contribute significantly to cancer progression. NETs may induce tumor cell proliferation by releasing factors that enhance the proliferative ability of cancer cells (42). They also contribute to the immunosuppressive tumor microenvironment by hindering the migration of cytotoxic immune cells and shielding tumor cells from immune-mediated killing (43). Furthermore, NETs play a role in awakening dormant cancer cells, promoting their proliferation and metastatic growth (44). By facilitating cell migration and metastatic microenvironment, NETs contribute to the overall progression of cancer (45).

The formation of NETs requires the presence of two essential proteins: integrin αvβ1 and matrix metalloproteinase 9 (MMP-9), which can capture and activate TGF-β (46). The activation of TGF-β triggers EMT in cancer cells and is associated with the progression of tumor cells (46, 47). The DNA component of NETs also plays a crucial role in tumors by interacting with receptors on tumor cells, influencing their behavior and contributing to the complex interplay between the immune system and cancer cells in the tumor microenvironment (48). Additionally, the chemical composition of NETs may also be a key factor, for example, IL-17 found in NETs can interact with cytotoxic CD8 T cells and exclude them from the tumor tissue (49).

## Tumor microenvironment in osteosarcoma

**Figure 3:**
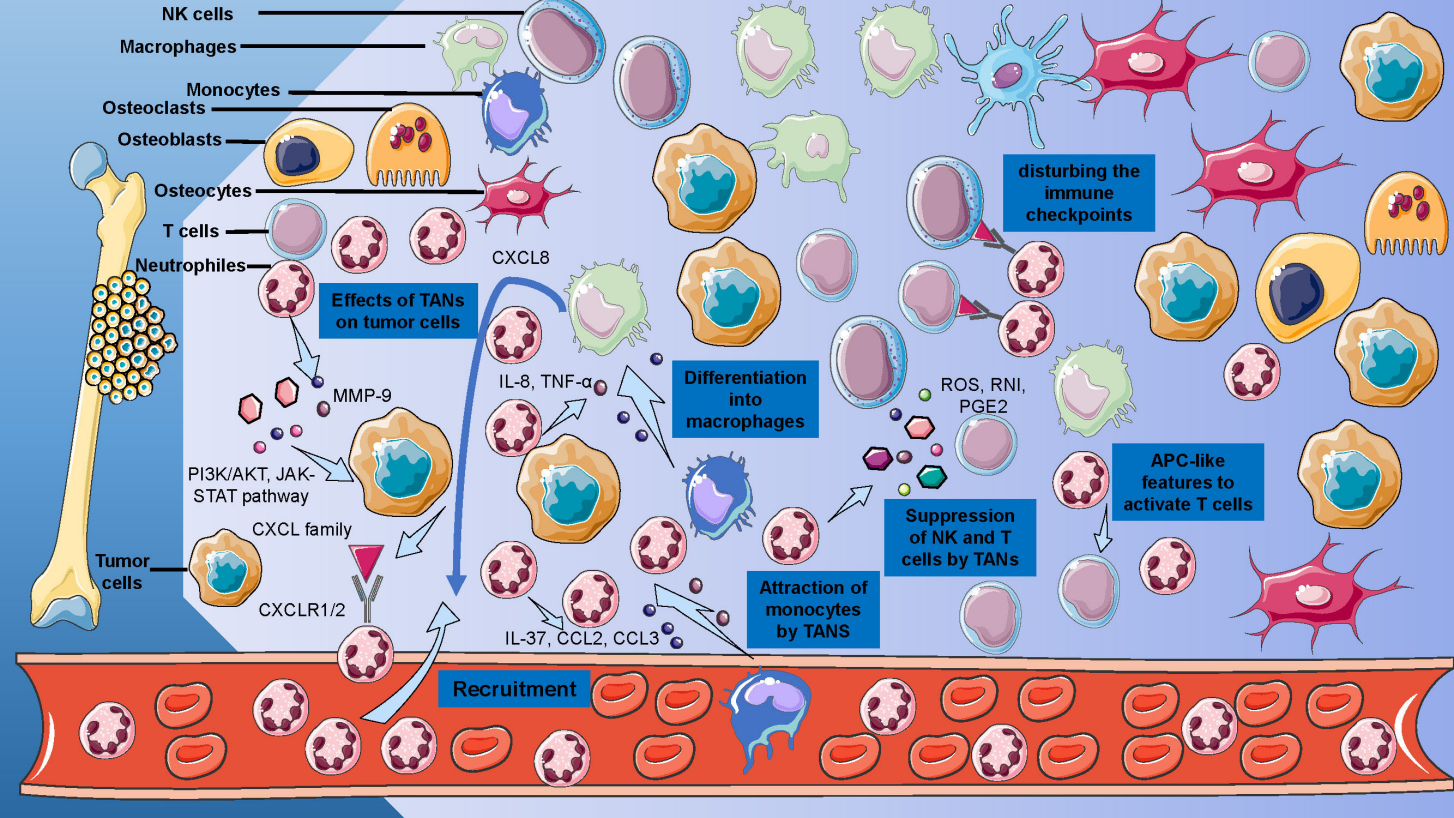
Cellular and Acellular Elements of the Osteosarcoma Tumor Microenvironment. Detailed representation of the osteosarcoma TME, delineates the interplay between osteoblasts, osteoclasts, MSCs, and immune cells, including T cells and macrophages.

The basis of understanding the roles of neutrophils in osteosarcoma is to decode the TME of osteosarcoma (**Figure 3**). The TME of osteosarcoma comprises a heterogeneous milieu of cellular components, including bone cells, stromal cells, vascular cells, immune cells, and the extracellular matrix (ECM) (5). Within the TME of osteosarcoma, interactions between tumor cells and stromal cells contribute to tumor growth, invasion, and metastasis (50). In this complex microenvironment, the immune system plays a paradoxical role: it may promote or suppress the progression of osteosarcoma, according to different TME and cellular phenotypes (51). Moreover, the ECM components in the TME of osteosarcoma provide structural support and signaling cues that influence tumor cell behavior, including migration, invasion, and drug resistance (52). Recent evidence also suggested that EVs, small membrane-bound vesicles released by cells into the ECM, may serve as a bridge of intercellular communication and metastasis (53).

In osteosarcoma, bone cells, including osteoblasts, osteoclasts, and osteocytes, play a crucial role in the TME (5). Osteoblast, a type of bone-forming cell, is originating from pluripotent mesenchymal stem cells. In the TME of osteosarcoma, osteoblasts may regulate the osteoclasts’ metabolism and communicate with osteosarcoma cells via multiple pathways, such as OPG/RANK/RANKL and Fas/FasL (54). Moreover, a recent study also reported that osteoblast may also regulate the TME by extracellular vesicles (55). Compared to osteoblasts, osteoclasts, cells derived from myeloid precursor cells and playing bone resorption effect, may play a more active area in osteosarcoma (50). From views of the population level, individuals with higher levels of osteoclast activity may have a lower risk of osteosarcoma and more satisfactory chemotherapy efficacy (56). Osteoclasts may activate CD4^+^ and CD8^+^ T cells and play an antigen-presenting cells-like role (57). However, in the different stages of osteosarcoma, osteoclasts may have different effects: in the early stage, osteoclasts may establish a niche containing osteosarcoma and suppress the metastasis, while in the later stage, accumulated tumor cells may have a stronger metastatic (58). Osteoclasts may also regulate the TME of osteosarcoma by interacting with CD4+ Tregs (59). Lastly, osteocytes as mature bone cells were also reported to have contributions to TME of osteosarcoma: osteocytes may have communications with osteosarcoma via the CXCL12-CXCR4 axis and by secreting TGF-β and VEGFA (60).

Another crucial cell in the TME of osteosarcoma is the mesenchymal stem cell (MSC), due to their potential roles as the precursors of osteosarcoma cells (61). The communications between MSCs and osteosarcoma cells have been reported in previous studies, and many factors, including CXCL12, IL-6, and VEGF, have been proven to be included (61). Moreover, extracellular vesicles may also mediate the interaction between MSCs and osteosarcoma cells by regulating the MALAT1/Wnt/β-catenin pathway and autophagy (62). Due to the characteristics and the roles of the extracellular vesicles from MSCs in the development of osteosarcoma, engineered extracellular vesicles may become a potential treatment for osteosarcoma by encapsulating drugs (63).

Recently, with the development of single-cell transcription, more understandings of immune TME of osteosarcoma are emerging. T cells play different roles in the TME of osteosarcoma due to the heterogeneity. Usually, CD8^+^T cells may directly attack tumor cells, and CD4 T cells may orchestrate the immunity, while Tregs act as an immune suppressor role (64). In the single-cell dynasty, the landscapes of T cells in osteosarcoma are more complex and diverse (13). T cells may also be regulated by TME. In TME, many chemokines, such as CXCL12, and many cell phenotypes, such as Tregs and myeloid-derived suppressor cells (MDSCs), may regulate the migration of T cells and contribute to the progression of osteosarcoma (13). Compared to T cells, B cells may mainly play tumor-promotion roles in osteosarcoma by secreting immune suppressive cytokines and activating Tregs (65). Recent studies have reported the potential checkpoint molecules on B cells, and targeting these checkpoints may become a potential strategy for osteosarcoma treatment (66). Another lymphoid cell in TME of osteosarcoma is the NK cell, a type of cell featured by its strong cytotoxic activity on malignant cells (67). NK cells may directly regulate the TME and establish the antitumor microenvironment by releasing IFN-γ (68). However, recent understandings from single-cell levels indicated that TME may also have an effect on NK cells and cause high levels of heterogeneity by regulating NK cell receptor signals and Tregs (13). Many studies have tried to develop potential methods based on NK cells to treat osteosarcoma, and these methods may enhance the anti-tumor effect of NK cells by targeting IL-12, IL-15, and so on (69).

Another immune cell lineage in osteosarcoma TME is monocyte lineage, including monocytes, macrophages, and dendritic cells (DCs). Monocytes play roles of antigen-presenting cells and could further differentiate into macrophages or DCs (70). Monocytes may release chemokine monocyte chemoattractant protein-1 (MCP-1), and MCP-1 may regulate the growth, metastasis, and progression of osteosarcoma cells (71). The recruitment of monocytes in TME is mainly regulated by CCL2, and by inhibiting the CCL2 receptor, the monocyte recruitment in TME may significantly decrease (72).

Monocytes may further differentiate into macrophages, which are the most abundant immune cells in the TME of osteosarcoma (73). Polarizing into M1 or M2 phenotype, macrophages may have contrast effects on tumor progression (74). M1 macrophages release proinflammatory cytokines, including nitric oxide synthase (iNOS), and tumor necrosis factor-alpha (TNF-α), and factors may lead to anti-tumor activity and induce the Th1 cells (75). Compared to M1 macrophages, M2 phenotype cells have contrasting activity: M2 macrophages exert their pro-tumor activities through various mechanisms in TME, including immune suppression, tissue remodeling, tumor progression, and angiogenesis (12). M2 macrophages promote osteosarcoma metastasis by secreting factors such as CCL18, MMP-12, COX-2, and IL-1β, and these factors may contribute to metastasis through the NF-κB/miR-181α-5p/RASSF1A/Wnt pathway (76, 77). M2 macrophages may also contribute to tumor angiogenesis by releasing vascular endothelial growth factor (VEGF) and fibroblast growth factor (FGF) (78). Recent studies also reported that M2 macrophages may inhibit the activity of T cells due to their expression of PD-1 (79). Repolarization of M2-like macrophages to M1-like macrophages is emerging as an innovative anticancer approach.

Another phenotype differentiated from monocytes is DCs, kinds of professional antigen-presenting cells (APCs) (68). By producing IFN-1α and IFN-1β, DCs may regulate anti-tumor effects (80). High levels of DCs may relate to high levels of heat shock promoter 70 (HSP70) and high activity of T cells (81). In view of results from single-cell RNAseq, DCs may have different phenotypes, and some of them may play immunosuppressive roles (82).

## Roles of neutrophils in osteosarcoma

The recruitment of neutrophils from the circulatory system to TME may undergo several stages, including attachment, adhesion, crawling, and transmigration (83). Chemokine of the CXC family is the major system that regulates the attraction of neutrophils, including seven chemokines and two receptors. Usually, the CXC family chemokines secreted by tumor tissues may attract the neutrophils by interacting with CXC receptors CXCR1 and CXCR2 expressed on neutrophils (84). After the receptor binding, the G-protein and β-arrestin signaling pathways were activated, which may further regulate the activation of calcium channels, phospholipase C, MAP, and tyrosine kinase pathways (85). The activation of these pathways may promote neutrophil migration by remodeling dynamic actin (86). Previous studies have reported that many neutrophil-regulating factors, such as CSF and IL-17, may regulate neutrophil attraction by regulating the levels of CXC chemokines and the receptions (87, 88). Many factors that play significant roles in neutrophil migration have been explored in osteosarcoma, including CXCL1, IL-6, CCL2, and so on (89). CXCL1 has higher expression in the tumor tissues compared to normal tissues, and the levels of CXCL1 expression may increase with the tumor progression, many factors were found to relate to this phenomenon, including the extracellular vesicles secreted by osteosarcoma cells, pH levels, and other cytokines released by tumor-relating cells (90, 91).

The recruitment of neutrophils to other organs is also significantly related to the formation of a pre-metastatic niche (92). Previous studies have proven that CXCL1 releasing by human pulmonary artery endothelial cells may significantly increase osteosarcoma cell mobility, and this phenomenon was mediated by VCAM-1 (93). Moreover, it was also reported that ANGPTL2 may contribute to the recruitment of neutrophils to the lung and promote the formation of lung pre-metastatic (92). Moreover, evidence from transcriptomic and histological analysis of premetastatic lungs has identified the characteristics of pro-metastatic events including inflammatory-induced stromal fibroblast activation, neutrophil infiltration, and ECM remodeling (94).

Though the research on TANs in osteosarcoma is still limited, the neutrophils and their related phenotypes have also been proven to have effects on the development and progression of osteosarcoma (95). In osteosarcoma, the N1 subtype of TANs may be more prevalent in the early stages of the disease, correlating with a favorable response to treatment and a better prognosis, while the N2 subtype could foster an immunosuppressive microenvironment that hinders effective immunotherapy (96). The unique behavior of neutrophils in osteosarcoma may be attributed to the bone matrix components and the specific cytokine milieu present in the OS microenvironment, which can influence neutrophil polarization and function (97).

Furthermore, recent multi-omics analyses identified distinct molecular subtypes of osteosarcoma, each with varying prognoses and responses to treatment (98). These subtypes exhibit different patterns of neutrophil infiltration and activation, suggesting that the regulatory mechanisms controlling neutrophil behavior may vary between osteosarcoma subtypes (98). TANs may regulate the development of osteosarcoma via matrix metalloproteinases -9 (MMP-9): the high levels of MMP-9 mediated by TANs are correlated with poorer prognosis (99). MMP-9 secreted from TANs may interact with insulin receptor substrate 1 (IRS-1), and further regulate the PI3K/AKT signaling pathway to contribute to the proliferation of tumor cells (100). From the insights of single-cell sequencing, the expressions of PPP2R5C, PPP2R5E, YWHAG, and CREBBP on TANs were significantly related to the metastatic of osteosarcoma, and these genes may play their roles via HIF-1, PI3K-AKT, and JAK-STAT signaling pathways (101). Similar to MMP-9, the PPP2R5C, a subunit of protein phosphatase 2A, was expressed on TANs, and may also regulate the proliferation of osteosarcoma via PI3K/AKT pathway (101). By comprehensively analyzing the significant genes from osteosarcoma and neutrophils at the single-cell level, hundreds of genes were identified, and C3AR1 and FCER1G as two neutrophil-related genes were validated to play critical roles in the communication between neutrophils and osteosarcoma cells (102). C3AR1 and FCER1G were highly regulated in the osteosarcoma mice induced by K7M2, and these two genes were proven to have significant prognostic value in osteosarcoma (102).

TANs may also act by communicating with other immune cells (103). The most significant cell phenotypes that relate to TANs are myeloid-derived suppressor cells (MDSCs), due to their shared origin (15). MDSCs, the immature myeloid cells, play significant roles in TME. In humans and mice, there are two major classes of myeloid-derived suppressor cells (MDSCs), classified based on their origins from the granulocytic lineage and monocytic lineage, namely polymorphonuclear-MDSCs (PMN-MDSCs) and monocytic-MDSCs (M-MDSCs) (104). The common feature of MDSCs is their appearance in immunologically activated pathological states, due to sustained stimulation of myeloid cells in environments such as cancer, chronic infections or inflammation, and autoimmune diseases, as a result of prolonged presence of myeloid growth factors and inflammatory signals. The main characteristic of MDSCs is their ability to suppress immune responses, including those mediated by T cells, B cells, and natural killer (NK) cells (105). M-MDSCs and PMN-MDSCs possess key biochemical features that contribute to immune response suppression, including upregulation of signal transducer and activator of transcription 3 (STAT3), induction of endoplasmic reticulum stress, expression of arginase 1, and expression of S100A8/A9 (106).

MDSCs may inhibit the migration of T cells and reduce T cell activity to protect osteosarcoma cells (107, 108). This function of MDSCs may be achieved by several pathways and factors, such as the production of nitric oxide (NO) and ROS, and the consumption of L-arginine (109). MDSCs may also contribute to the metastasis of osteosarcoma by forcing T-cell tolerance and releasing TGF-β and hepatocyte growth factor (HGF) (110). Moreover, MDSCs may also regulate tumor angiogenesis by releasing VEGF and MMP-9 (111).

Another remarkable cell interaction with neutrophils is tumor-associated macrophages (15). The neutrophils may attract monocytes by secreting IL-37, CCL2, and CCL3, and these monocytes may differentiate into macrophages via IL-8 and TNF-α (112). It was also reported that in the development of sarcoma, TANs may regulate the IL-12 releasing of macrophages, and IL-12 may contribute to the activation of unconventional T cells due to their high levels of IL-12R expression, which further regulate the secreting of IFN-γ and tumor suppression (113). During the nascent stages of oncogenesis, macrophages exhibit tumoricidal properties due to their activated state, generating reactive oxygen and nitrogen species that can induce DNA damage and genetic instability (114). The cytokines from neutrophils in the tumor microenvironment may significantly impact macrophage functions and phenotypes (115). Furthermore, macrophages may contribute to malignant transformation through the secretion of angiogenic factors, proteases, and growth factors (116). These factors stimulate cancer cell proliferation and support the epithelial-mesenchymal transition in tumor cells, thereby facilitating tumor growth and metastasis (117). Recent studies also reported that these recruited monocytes and macrophages may release CXCL8 to further attract neutrophils, which may become a feedback loop (118).

TANs may also interact with lymphoid cells and have paradoxical effects on the functions of lymphoid cells (119). TANs may release ROS, reactive nitrogen intermediates (RNI), and prostaglandin E2 (PGE2), and these factors may directly inhibit the functions of T cells and NK cells (120, 121). The release function of TANs may relate to their metabolism status. Facing limited glucose supply, neutrophils may have high levels of mitochondrial fatty acid oxidation and high ROS production (120). Moreover, TANs with endoplasmic reticulum stress and altered lipid metabolism may also have higher levels of ROS production (122). In addition to the release of mediate factors, TANs may also interact with lymphocytes by disturbing the immune checkpoints, due to the expression of PD-L1 and VISTA on neutrophils, which may result in the dysfunction of T cells and NK cells by interacting with their ligands (123). It was also reported that some types of neutrophils may directly contact CD4+ T cells physically to inhibit the functions of the cells (124). TANs may regulate the activity of T cells by attracting Tregs and formatting the TME. Interestingly, TANs may also have positive effects on lymphoid cell activation. TANs may activate T cells by showing their APC-like features, and these APC-like features in TANs are activated by TME-derived CSF and IFN-γ (125). Furthermore, the activation of T cells may contribute to the expression of CD54 and CD86 on TANs, which may further strengthen the APC-like features of TNAs and construct a positive feedback loop (126). By secreting IL-1β and IL-18, neutrophils also directly attract and activate NK cells (127).

Within the context of the tumor microenvironment, the intricate interplay between neutrophils and B cells holds substantial implications for cancer progression and therapeutic strategies (124). Neutrophils have been shown to facilitate the migration of B cells through the release of TNF-α, with this effect being notably enhanced by the presence of specific chemokines, including CXCL13 and CXCL12 (128). While the precise nature of the interaction between neutrophils and follicular B cells remains to be fully elucidated, it is observed that neutrophils tend to concentrate in areas rich in B cells and secrete B-cell-activating factor (BAFF) under the influence of G-CSF, which in turn, bolsters the rapid production of plasma cells (129). Furthermore, neutrophils are known to regulate immunoglobulin production by interacting with the BAFF receptor on B cells, a pivotal mechanism in the modulation of the humoral immune response (130). This capability of neutrophils to influence B cell activity is particularly significant when considering the diverse functions of B cells in countering tumorigenesis and their ability to stimulate other immune cells, including T and NK cells (131).

The roles of NETs in osteosarcoma were also reported in previous studies. In the osteosarcoma gene profiles, more than 90 NETs genes were identified, and these genes were related to immune cell infiltration, including NK cells and CD8+ T cells (8). Previous studies tried to establish a prognostic signature based on NETs-related genes to predict the overall prognosis of osteosarcoma and proved the strong performance of this signature (11). Similarly, the TME between groups with different levels of NETs-related signatures may have different types of immune cell infiltration.

Though few functional and experimental studies tried to explore the specific mechanism of NETs in osteosarcoma, the normal function and features of NETs in general tumors may provide us with a potential hypothesis. In the context of tumor development, NETs may serve to limit tumor spread in the early stages by directly entrapping and killing cancer cells (132). Moreover, NETs can enhance the local immune response by promoting the recruitment and activation of immune cells such as T cells and natural killer (NK) cells. This can lead to the secretion of cytokines and chemokines that reinforce the inflammatory response and potentially contribute to the elimination of cancer cells (133). Generally, some NETs-related factors, such as IL-8, G-CSF, and CXC chemokine receptor family, have been also proven to relate to the progression of osteosarcoma (134). Similarly, MMP-9, mentioned many times in this review, as a critical protein of NETs, is also proven as a key factor in osteosarcoma (135). In addition, the roles of NETs in blocking immune cells and protecting cancer cells physically may also exist in the TME of osteosarcoma. The overall roles of neutrophils in osteosarcoma are summarized in **Figure 3**.

## Clinical evidence of neutrophils in osteosarcoma

Previous studies reported the neutrophil count was an independent risk factor for the metastasis of osteosarcoma (136). However, the most widely used predictive parameter related to neutrophils in osteosarcoma is the neutrophil-to-lymphocyte ratio (NLR), which may predict many kinds of prognoses, including overall survival, progression-free survival (PFS), disease-free survival (DFS), metastasis, and so on (137–139). In a cohort enrolling 359 individuals after surgeries for osteosarcoma, pre-treatment NLR may independently predict the overall survival and PFS: the individuals with higher NLR may have lower 5-year overall survival (HR = 1.80, 95% CI = 1.35-2.41, P < 0.001) and PFS (HR = 1.65, 95% CI = 1.26-2.15, P < 0.001) compared with those with low levels of NLR (137). Similar results were also reported in a study that included 100 children with osteosarcoma, rhabdomyosarcoma, and Ewing sarcoma: the NLR > 2 may independently predict the overall survival (HR = 2.27, 95% CI = 1.07-5.30, P = 0.046) for children with osteosarcoma (10). Compared to other hemogram parameters, such as platelet-to-lymphocyte ratio (PLR) (AUC = 0.668 and AUC = 0.600) and lymphocyte-to-monocyte ratio (LMR) (AUC = 0.609 and AUC = 0.407), NLR (AUC = 0.749 and AUC = 0.663) has the highest predictive value for overall survival (140, 141). Pre-treatment NLR may also predict the efficacy of neoadjuvant chemotherapy in osteosarcoma, and the results from multicenter cohorts showed that the patients with lower NLR may be more likely to achieve pathological complete response (OR = 2.82, 95% CI = 1.36-5.17, P = 0.020) compared with patients with high NLR (142). Similarly, a cohort from Iran with 186 individuals also reported that the pre-treatment NLR may effectively predict the response after neoadjuvant chemotherapy and overall survival: the patients with high NLR have significantly low overall survival (20.7 months vs. 34.6 months, P = 0.003) and DFS (20.4 months vs. 32.7 months, P = 0.020) compared with individuals with normal NLR (143). The prognostic abilities of increased NLR for overall survival (HR = 1.30, 95% CI = 1.10-1.50, P = 0.002) were also reported in individuals with osteosarcoma and treated with high-dose methotrexate and etoposide/ifosfamide chemotherapy (144). Beyond the pre-treatment NLR, a recent study also reported the prognostic value of dynamic changes of NLR during the treatment: by combining the baseline NLR and Delta NLR, the NLR staging system (HR = 2.46, 95% CI = 1.63-3.71, P < 0.001) may have better predictive values (145).

In addition to the simple immune inflammation index such as NLR, many immune indices related to neutrophils were also reported to be used to predict the outcome of osteosarcoma. For example, the systemic immune inflammation index (SII), defined as platelet × neutrophil/lymphocyte counts, was reported to relate to tumor size, histological type, Enneking stage, and neoadjuvant chemotherapy, and high SII (HR = 1.22, 95% CI = 1.10-1.45, P = 0.029 and HR = 1.01, 95% CI = 1.00-1.02, P = 0.015) may independently predict the overall survival (146, 147). A multicenter study also reported the prognostic values of pre-operative SII in the overall survival of both young (≤20 years) individuals (HR = 2.38, 95% CI = 1.02-5.56, P = 0.045) and older (60-80 years) individuals (HR = 2.42, 95% CI = 1.03-5.68, P = 0.043) with osteosarcoma (148). In addition to SII, the lung immune prognostic index (LIPI), calculated by serum lactate dehydrogenase (LDH) and neutrophil to lymphocyte ratio (NLR), was also proven to predict the metastasis (HR = 1.864, 95% CI = 1.11-3.13, P = 0.018) of osteosarcoma (149). Moreover, studies included 133 individuals with osteosarcoma reported that the pre-treatment Naples prognostic score (NPS), composed of serum albumin level, serum total cholesterol (TC), lymphocyte-to-monocyte ratio (LMR), and neutrophil-to-lymphocyte ratio (NLR), was able to predict the overall survival (HR = 5.87, 95% CI = 1.03-6.43, P < 0.001; HR = 6.55, 95% CI = 1.15-13.62, P < 0.001) and PFS (HR = 5.27, 95% CI = 1.02-11.49, P < 0.001; HR = 6.78, 95% CI = 1.23-10.58, P < 0.001), and was significantly related to tumor location (P = 0.009), Enneking stage (P < 0.001), pathological fracture (P = 0.005), local recurrence (P < 0.001), and metastasis (P = 0.003) (150). The studies related to the clinical roles of neutrophils are summarized in **Table 1**.

**Table 1:** Studies focusing on the clinical roles of neutrophils in osteosarcoma.

Index	Sample size	Location	Population Characteristics	Study type	Outcome	Conclusion	Ref
Pretreatment Neutrophil count	65	Japan	First visit osteosarcoma patients without metastasis	Single-center retrospective study	Metastasis	Low neutrophil count as a risk factor for metastasis of osteosarcoma	(122)
Pretreatment NLR	359	China	Patients who underwent curative surgery for osteosarcoma	Single-center retrospective study	5-years OS/PFS	High levels of NLR as risk factors for survival of osteosarcoma	(123)
NLR	2087	–	Patients with osteosarcoma	Meta-analysis	OS/DFS	High levels of NLR as risk factors for survival of osteosarcoma	(124)
NLR	2162	–	Patients with osteosarcoma	Meta-analysis	OS/DPS	High levels of NLR as risk factors for survival of osteosarcoma	(125)
Pretreatment NLR	172	Turkey	Pretreatment patients with osteosarcoma	Single-center retrospective study	OS	High levels of NLR as risk factors for survival of osteosarcoma	(126)
Pretreatment NLR	162	China	Pretreatment patients with osteosarcoma	Single-center retrospective study	OS	High levels of NLR as risk factors for survival of osteosarcoma	(127)
NLR at the first cycle of chemotherapy	96	China	Patients who underwent NACT for osteosarcoma	Multi-center retrospective study	pCR	High levels of NLR as risk factors for the effect of NACT	(128)
NLR at the first cycle of chemotherapy	186	Iran	Patients who underwent NACT for osteosarcoma	Multi-center prospective study	OS/DFS	High levels of NLR as risk factors for survival of osteosarcoma	(129)
NLR during chemotherapy	164	France	Patients with osteosarcoma and treated with M-EI chemotherapy	Multi-center prospective study	OS/EFS	High levels of NLR at 4 weeks as risk factors for survival of osteosarcoma	(130)
NLR (baseline, during treatment, delta)	251	China	Pretreatment patients with osteosarcoma	Single-center retrospective study	OS	High levels of baseline NLR and delta NLR as risk factors for survival of osteosarcoma	(131)
Pretreatment SII	126	China	Pretreatment patients with osteosarcoma	Single-center retrospective study	OS	High levels of SII as risk factors for survival of osteosarcoma	(132)
Pretreatment SII	86	China	Pretreatment patients with osteosarcoma	Single-center retrospective study	EFS/CSS	High levels of SII as risk factors for survival of osteosarcoma	(133)
Pretreatment SII	125	China	Pretreatment patients with osteosarcoma	Single-center retrospective study	OS	High levels of SII as risk factors for survival of osteosarcoma	(134)
Pretreatment LIPI	184	China	Pretreatment patients with osteosarcoma	Single-center retrospective study	Metastasis	Low levels of LIPI as risk factors for metastasis of osteosarcoma	(135)
Pretreatment NPS	133	China	Pretreatment patients with osteosarcoma	Single-center retrospective study	OS/PFS	High levels of NPS as risk factors for survival of osteosarcoma	(136)

## Neutrophils in the surgery of osteosarcoma

**Figure 4:**
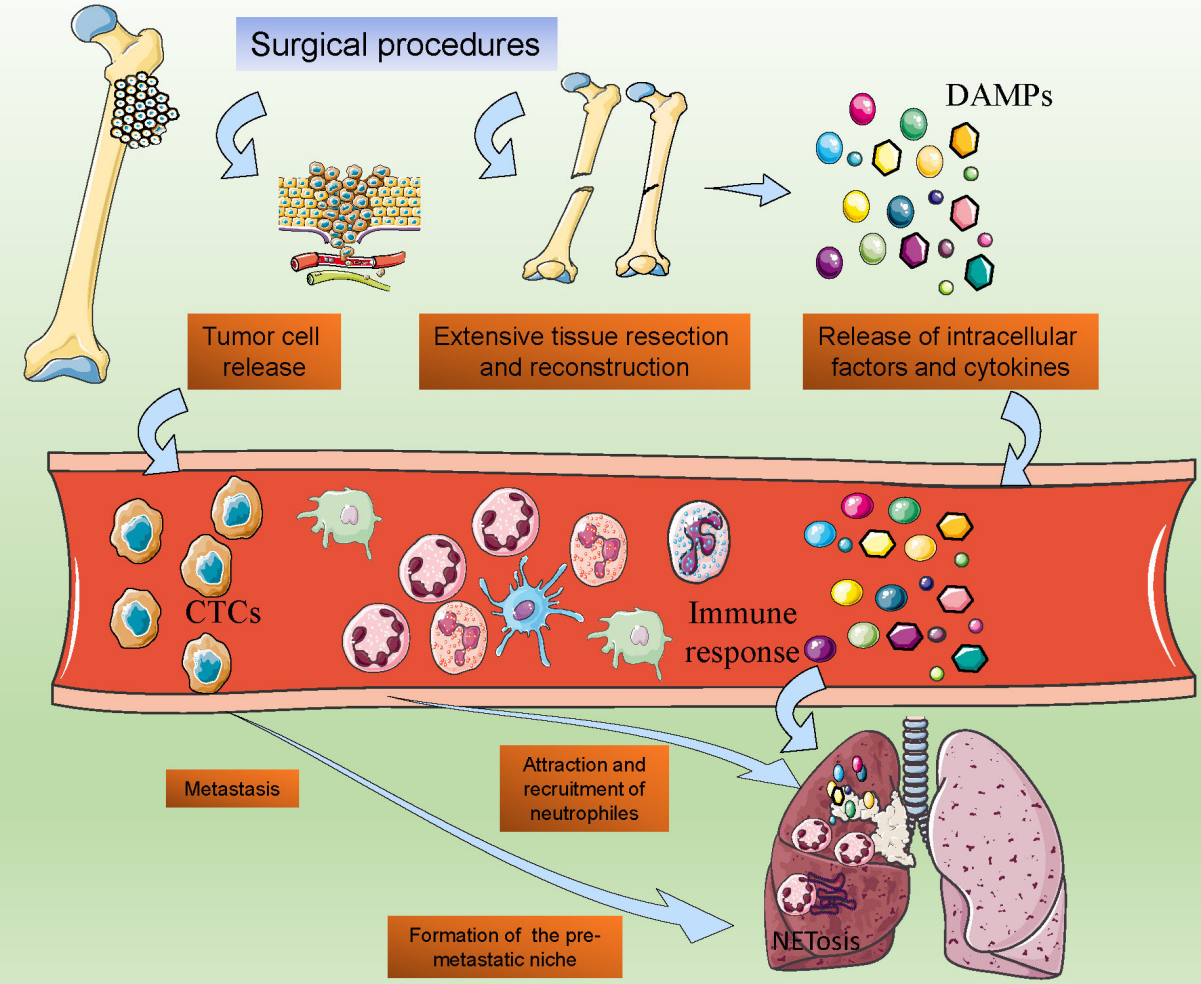
Post-surgical Neutrophil Activation and Its Implications in Osteosarcoma Metastasis. The post-operative surge in neutrophil activity following osteosarcoma surgery, showcasing the release of damage-associated molecular patterns (DAMPs) and subsequent neutrophil chemotaxis. It suggests a model where surgical stress-induced NETosis and the formation of pre-metastatic niches in distal organs are potential drivers of tumor cell dissemination.

In the treatment of osteosarcoma, surgical resection is the most critical treatment strategy. However, even after the surgical procedure, patients with osteosarcoma may also face a high risk of postoperative metastasis (151). Recent studies reported that the postoperative metastasis was partly driven by the immune response caused by infection, tissue damage, and cell injury, and the surgical procedure of osteosarcoma is the major cause, due to the extensive tissue resection and reconstruction (152). Even after surgery, the tissue healing process also activates systemic inflammatory reaction, which establishes a favorable microenvironment for tumor growth and metastasis (153). Neutrophils may play critical roles in this kind of acute inflammatory response (**Figure 4**).

After the surgical procedures of osteosarcoma, the extensive trauma caused by surgery may result in high levels of release of intracellular factors and cytokines, which may initiate the immune response and naturally increase the circulation neutrophil counts (154). The damaged cells after surgery expressed increased levels of damage-associated molecular patterns (DAMPs), groups of cellular components including ATP, DNA, cytokines, and so on (155). DAMPs in the local tissues may attract the circulation of neutrophils and contribute to the activation of neutrophils (116). A recent study reported that with the cell destruction, mitochondrial DNA (mtDNA) was released into circulation, and attracted neutrophils to format the pre-metastatic niche, which indicated the roles of neutrophils in the surgery-induced osteosarcoma metastasis (156).

Facing the extensive simulation, the NETosis of neutrophils would be activated. Previous studies have proven that after major surgeries, especially the large removal and reconstruction in tumor surgeries, the NETs markers in circulation may significantly increase (157, 158). Additionally, the intraoperative surgical vascular occlusion and hypoxia in the surgery procedures may also contribute to NETosis (159). The extensive release of NETs may finally promote the metastasis of tumors and result in the failure of radical surgery.

## Potential treatments based on neutrophils

**Figure 5:**
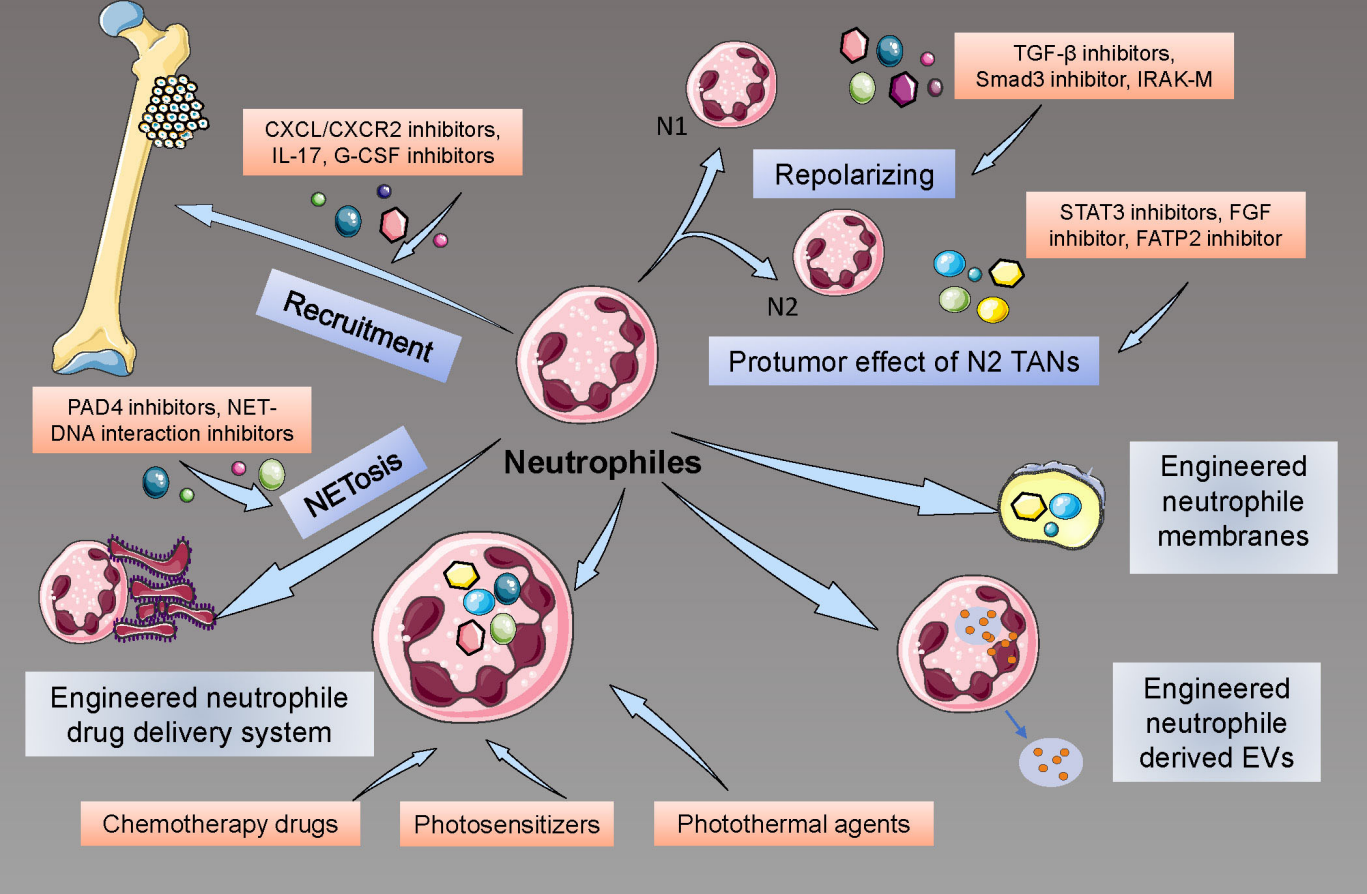
Therapeutic Interventions Targeting Neutrophils in Osteosarcoma. These include neutralizing chemokine gradients that recruit neutrophils to the TME, pharmacological repolarization of TAN phenotypes using TGF-β inhibitors, and the innovative use of engineered neutrophils for precision drug delivery, highlighting the potential of these approaches to disrupt osteosarcoma progression and enhance therapeutic responses.

How to benefit the patients more by targeting neutrophils? Many researchers have begun their explorations in the engineering and targeting of neutrophil strategies. Due to the limited recognition of the neutrophils in the cancer, especially in osteosarcoma, few studies about the neutrophil treatment in osteosarcoma have been reported. Here, we reviewed the progress and explorations in targeting and engineering neutrophils for cancer treatment to provide potential ways to treat osteosarcoma (**Figure 5**).

The recruitment of neutrophils in local tissues plays a significant role in tumor development and metastasis, so many studies tried to inhibit this process to achieve the goals of cancer therapy. Targeting CXCL/CXCR2 signaling has been reported as a potential treatment in cancer: by regulating neutrophil infiltration, CXCR2 inhibition, and interference may significantly suppress the tumor growth and prolong the survival of mice with tumor, as well as improve the quality of chemotherapy (160, 161). In addition to the direct effects on tumor development, anti-CXCR2 may also improve the effectiveness of PD-1 strategies, suppress the inflammatory microenvironment caused by neutrophils, and inhibit the formation of NETs (162). Similarly to CXCR2-related strategies, targeting CXCR4 may also have effects on tumor development by disturbing the recruitment of neutrophils (163). Additionally, many cytokines, proteins, and novel nanomaterial drugs, such as CEMIP (cell migration-inducing protein), IL-17, G-CSF-inhibiting antibodies, and colchicine, were reported to have a potential suppressive effect on neutrophil recruitment and have potential to become a treatment of cancers (164–166).

Due to the different phenotypes of TANs, researchers also tried to develop methods to repolarize pro-tumor TANs to anti-tumor TANs. As the TGF-β/Smad pathway is critical to the polarization of N2 TANs, some studies explored the potential strategies that may inhibit this signal, and usage of TGF-β receptor inhibitor and Smad3 inhibitor, as well as knockdown of Smad3, can effectively contribute to the repolarization to N1 phenotypes and enhance the anti-tumor effect of neutrophils (167). Similarly, enhancing the pathways that contribute to the polarization of TANs to anti-tumor phenotypes may also provide a potential effect on tumor suppression, such as interferon therapy (22). Recently, many new factors that may affect the repolarization of TANs were also reported, including Interleukin-1 receptor-associated kinase M (IRAK-M), nicotinamide phosphoribosyltransferase (NAMPT), novel TGF-β inhibitor, and proteins for inflammation resolution (168–170).

Trying to eliminate the effects caused by pro-tumor TANs may also be a potential intervention in targeting neutrophils for cancer treatments. Studies reported that by using STAT3 inhibitors, the activation of neutrophils was suppressed and turned into an anti-tumor phenotype (171). Targeting the angiogenesis effect of neutrophils may also inhibit the development and metastasis of tumors (172). A recent study also proved that inhibiting the FGF pathway could eliminate the neutrophil-activated effect on tumor cells (173). Moreover, many factors and proteins were found to have a regulatory impact on neutrophil activity by inhibiting specific pathways, such as fatty acid transport protein 2 (FATP2) inhibitor, HDAC inhibitor, leukotriene-generating enzyme arachidonate 5-lipoxygenase (Alox5) inhibitor, and so on (174, 175).

Targeting NETs is also a potential strategy. Targeting the protein structures of NETs, previous studies tried to use DNase I and (protein-arginine deaminase 4) PAD4 inhibitors, as well as laminin antibodies, to inhibit the protective roles of NETs in tumor progress (176). Due to the unstable nature of these inhibitor proteins, some nanomaterials were designed to precisely release the protein inhibitors at the accurate tumor location, which has achieved satisfactory effects (177). Similarly, studies also reported the exploration of targeting NETs-DNA. By designing cationic materials that may inhibit the interaction between NETs-DNA and CCDC25, researchers significantly reduced the infiltration of NETs-DNA and suppressed the metastasis of tumors (178).

Based on the characteristics of neutrophils that neutrophils may release cargo in certain microenvironments, engineering neutrophils were also considered as a potential treatment for tumors. Recent studies have reported that neutrophils may be engineered as cell drug delivery systems to transport chemotherapy drugs, photosensitizers, photothermal agents, and so on to tumor tissues efficiently and safely (179, 180). Neutrophil membranes have also been engineered for novel therapy strategies for tumors. The cell membranes derived from different cells have similar structures and functions to their derived cells, which were considered to have the potential to interact with tumor cells and to deliver drugs accurately (181). Recent studies designed the neutrophil membranes loaded celastrol (CLT), paclitaxel (PTX), and so on to treat cancer and achieved significant anti-tumor efficacy (182, 183). It was also proven that irreversible electroporation may induce the attraction of neutrophils and then improve the drug delivery effectiveness mediated by neutrophils (184). Additionally, neutrophil-derived extracellular vesicles (EVs) were also considered as potential platforms for drug delivery. Due to the inflammatory chemotaxis of neutrophil-derived EVs, EVs may automatically migrate to the inflammatory site, as well as tumor location (185). Engineered neutrophil-derived EVs may directly kill tumor cells and regulate TME by carrying drugs, miRNAs, and cytotoxic factors such as doxorubicin, granzyme, perforin, and so on (186, 187).

In addition to normal engineered neutrophil systems, many other engineered strategies based on neutrophils have also been developed. A recent study developed a two-pronged delivery system to inhibit the effect of neutrophils in TME by both eliminating NETs and reducing mitochondrial biogenesis (188). The design of this strategy was based on the positive feedback loop that hypoxia caused by exceeding mitochondrial activity may promote the formation of NETs and NETs may positively contribute to the mitochondrial metabolism. It was also reported that a drug delivery system based on a platelet-neutrophil hybrid Membrane may achieve efficient drug delivery guided by neutrophil-related inflammatory microenvironment and enhance the anti-tumor effect of macrophage (189).

However, the challenges and potential side effects associated with neutrophil-related therapies should also be noticed. Usually, chemokine inhibitors and chemokine receptor inhibitors were usually employed to achieve the strategies targeting on the recruitment, repolarization, pro-tumor effect, and NETosis (162). The human chemokine system is characterized by its intricate and diverse nature. The inhibition of a pivotal chemokine receptor could potentially result in significant adverse effects (72). Moreover, the redundancy inherent in chemokines and their receptors necessitates the use of appropriate initiating doses and metabolic stabilizers for the antagonists to be effective. This requirement significantly constrains the development of chemokine receptor antagonists and their clinical efficacy (190). Consequently, there is a pressing need to optimize chemokine receptor antagonists in future research and development endeavors. Moreover, we have to further explore the effects of neutrophil subtypes. As neutrophils play a critical role in innate immunity, broad targeting of these cells can increase the risk of infections and other diseases (98). Therefore, therapies must be tailored to minimize off-target effects on normal neutrophil functions. Engineered neutrophil systems may achieve more satisfactory effect due to their ability in precision targeting, immunomodulation, and rapid response (187). They can be designed to deliver drugs directly to the tumor site, increasing the efficacy of chemotherapy and reducing systemic side effects (173). Despite these advantages, their stability within the complex *in vivo* environment can be a concern, potentially affecting their therapeutic efficacy. Ensuring biocompatibility to avoid adverse immune responses is also a significant hurdle (173).

In summary, while neutrophil-based therapeutic strategies show promise in the fight against cancer, they also present significant scientific and technical challenges. Future research must focus on overcoming these hurdles, refining these therapies to maximize their efficacy and minimize adverse effects, and identifying biomarkers that can predict treatment response. This will be crucial in translating these innovative approaches into clinical practice, offering new hope for cancer patients.

## Challenges and perspectives

With the development of single-cell sequencing, the roles of neutrophils in tumor development and metastasis are gradually recognized and understood. However, their involvement in bone cancer, specifically osteosarcoma, remains relatively understudied compared to other types of tumors. Recent research has partly demonstrated the roles of neutrophils in osteosarcoma, but the specific roles of neutrophils in osteosarcoma, including the interaction with bone cells (osteoclasts and osteoblasts), the communication with immune cells, and the effects of NETs, were still not fully understood. Moreover, further engineering and targeting therapies based on neutrophils were still limited.

In the future, further research is needed to elucidate the complex interplay between neutrophils and tumor cells in osteosarcoma, as well as to identify novel therapeutic targets to modulate neutrophil function and improve patient outcomes. Single-cell transcriptomics allows for a detailed analysis of the transcriptomic profiles of neutrophils in different osteosarcoma subtypes (191). By comparing the gene expression profiles of N1 and N2 neutrophils in various osteosarcoma subtypes, we can gain insights into the molecular mechanisms underlying their polarization and function. This can be optimized by using advanced computational methods to analyze the single-cell data and by integrating the data with proteomic and metabolomic profiles. Moreover, given the role of exosomes and other extracellular vesicles in cell-cell communication, future research should focus on their role in neutrophil-bone cell interactions (187). Isolation and characterization of these vesicles from osteosarcoma subtypes can reveal novel biomarkers and therapeutic targets. Additionally, the development of preclinical models that accurately recapitulate the bone tumor microenvironment will be essential for advancing our understanding of neutrophil biology in osteosarcoma and translating these findings into clinical practice. Many models in bone metabolism research may achieve the regulation of different bone cells, such as B-hRANKL mice, B-hSOST mice, B-hRSPO1 mice, and so on (192). Integrating the bone-related diseases models, orthotopic osteosarcoma models and neutrophil models, we may further explore the potential mechanism of the neutrophils in osteosarcoma.

## Conclusion

In this review, we tried to summarize the roles of neutrophils in osteosarcoma from various dimensions, including the NETs, TANs in immune TME, interaction between neutrophils and immune cells, clinical evidence of neutrophils in osteosarcoma, the roles of neutrophils in surgery, and the potential therapy based on neutrophils. Though the studies on neutrophils in osteosarcoma were still limited, taking inspiration from studies on neutrophils in other types of cancer can also provide valuable insights for future research on their role in osteosarcoma development.
